# Monitoring a keystone species (*Alosa pseudoharengus*) with environmental effects: A comparison with direct capture and environmental DNA

**DOI:** 10.1371/journal.pone.0324385

**Published:** 2025-05-23

**Authors:** Matthew M. Dougherty, Andrew MacDonald, Geneva York, David M. Post

**Affiliations:** 1 Ecology and Evolutionary Biology, Yale University, New Haven, Connecticut, United States of America; 2 Biology, Saint Norbert College, De Pere, Wisconsin, United States of America; 3 School of Marine Sciences, University of Maine, Orono, Maine, United States of America; University of Hyogo, JAPAN

## Abstract

Keystone species are important drivers of ecological processes. Their ecological importance makes them prime candidates for biological monitoring, both to preserve and restore their populations when facing decline, and to limit their spread as invasive species. To monitor species well requires cost and labor efficient methods that are capable of detecting the target species at low abundances. Traditional sampling methods, or methods of direct capture, can be labor intensive when trying to monitor large areas or species at low abundances. Another method, environmental DNA (eDNA), has emerged as a more cost and time efficient supplement to traditional monitoring methods. Environmental DNA techniques and strategies continue to be developed, but face limitations for some taxonomic groups within certain habitats. Here, we propose a novel method for monitoring keystone species: environmental effects sampling. Keystone species have large effects on their environment relative to their abundance. Measuring their environmental effects—or quantifiable changes in the biotic or abiotic environment due to organism-environment interactions—has potential as a low-effort and low-cost method for detecting keystone species. In this study, we compare the effectiveness of traditional sampling, eDNA methods, and environmental effects sampling as an alternative low cost and time efficient method for monitoring the presence and abundance of an ecologically important keystone species, the alewife, *Alosa pseudoharengus*, in freshwater lakes. The alewife is a zooplanktivorous fish managed as a species of conservation concern along coastal New England, USA, and an invasive or non-native species throughout the Laurentian Great Lakes watershed. We sampled lakes throughout Michigan and Connecticut from 2018–2020 and compared the three monitoring methods along four axes: alewife presence/absence, alewife abundance, financial cost, and time efficiency. Our results suggest that monitoring alewife with environmental effects is more accurate, more cost efficient, and more time efficient than purse seining and eDNA. Our environmental effects results also led to the discovery that two historically recognized alewife lakes no longer contained alewife, as confirmed by traditional sampling. However, environmental effects monitoring was only useful for determining alewife presence/absence, and was not reliable for determining alewife relative abundance. Environmental effects monitoring presents novel opportunities for efficiently and effectively monitoring keystone species such as alewife for the purpose of restoration or management.

## Introduction

Biological monitoring is important to the conservation and management of a wide range of species and ecosystems [1, 2, 3]. Monitoring keystone species, or species with an outsized influence over the environment relative to their abundance, is of special importance because of their large ecological impacts [4, 5, 6]. Keystone species drive important ecological processes, exhibit low functional redundancy, and can have significant cultural and economic impacts even at relatively low densities [7, 8, 9]. The influence of keystone species often makes them a target of restoration efforts when their populations are in decline, a target of management for eradication, or to limit their spread when invading new territories [10,11]. Therefore, effective monitoring of keystone species requires detection at low abundances, and can require high intensity sampling [3].

Traditional sampling uses methods that directly measure the presence of an organism within an environment. When sampling for fish, traditional sampling often takes the form of nets, hook and line, or electrofishing equipment [see 12, 13, 14]. Traditional sampling can be reliable for many species, especially when it is cheap, requires little labor, and can detect species presence at low abundances [3]. However, such monitoring projects can be difficult to maintain due to cost, labor efforts, and context and species-specific demands, especially when monitoring large areas with few resources. It is essential to discover and develop cost-effective, low effort tools for effective ecological monitoring, and therefore ecosystem management.

Environmental DNA (eDNA) has emerged as a promising tool for more effective species monitoring [15,16]. eDNA describes a method for detecting and identifying organisms from small traces of DNA found in environmental samples, such as water, soil, or air [17]. When interacting with an environmental substrate, organisms leave behind material containing DNA, allowing researchers or managers to identify the species by analyzing samples of the substrate with molecular methods. eDNA has been used to successfully detect various species—including bacteria [18], plants [19], invertebrates [20], birds [21], amphibians [22], fish [23], mammals [24], reptiles [25], among others—in a variety of ecosystems, and sometimes at low abundances [20,26]. Environmental and organismal covariates to species abundances that could influence the effectiveness of eDNA have been widely studied, including the importance of temperature, sunlight, DNA persistence and degradation, the target organism’s relative abundance, and the target organism’s life stage [20,27–29].

However, eDNA is not always the most effective or efficient tool for species monitoring [30]. For example, species with exoskeletons do not have mucous producing epidermal cells, and therefore may not release DNA into the environment to the same extent as fish and amphibians, weakening the efficiency of eDNA methods for those species [30, 31, 32]. Aquatic organisms that reproduce asexually may release less DNA than sexually reproducing organisms that release sperm into an environmental substrate [32]. Likewise, strong seasonal population dynamics of the target species, or regular disturbance of the environment, may also make eDNA ineffective some of the time [30].

We propose monitoring keystone species through the quantification of their environmental effects as an alternative or supplement to traditional sampling and eDNA. Because of their large per-capita influence over the environment, many keystone species may be detected at low abundances *indirectly* by sampling for their keystone environmental effects. Environmental effects can include any quantifiable change to the environment due to organism-environment interactions. For example, organisms could be detected by the presence of artifacts they leave behind. Beavers could be detected by their dams, [33] and termites by their mounds [34]. Environmental effects can also include consumption effects. Pisaster sea stars could be monitored by the presence or abundance of mussels, their preferred prey [35], or sea otters by the presence and abundance of kelp [36]. However, to our knowledge, no study has recommended using environmental effects as a practical tool for keystone monitoring, including for the examples listed above. Studies comparing traditional methods, eDNA, and environmental effects as monitoring tools are needed to better inform managers and researchers about how to best monitor keystone species.

The alewife, *Alosa pseudoharengus,* is a keystone species that is a good model for testing the relative strengths and weaknesses of these monitoring methods. An ecologically and economically important fish in North America, [8,37–39], alewife are a keystone species within freshwater lakes, and their environmental effects have been well studied for decades [40,41,43–46]. Alewife are voracious foragers that eat almost 40% of their body weight in zooplankton daily as juveniles [42]. They dramatically reduce the size structure of zooplankton communities through size-selective foraging, giving them an outsized influence on their environment relative to their abundance [40,41].

The alewife is one species that exhibits two distinct life history forms with different implications for conservation and management, and each form is sampled with different methods [41; 43;47]. Anadromous alewife migrate between marine and freshwater environments to spawn and are listed as a species of conservation concern [48]. Anadromous alewife are managed to increase their density and access to spawning habitat. They are often monitored in streams as adults with electronic fish counters or by volunteers who manually count fish as they migrate from the ocean into freshwater lakes to spawn [38,49,50]. Landlocked alewife spend their entire life cycle within freshwater and are considered non-native or invasive species in many regions of the United States, and are managed to prevent their spread while minimizing their impact and maximizing their value to fisheries [8,51]. Landlocked alewife are sampled with purse seines in small lakes or trawls in large lakes to determine presence and estimate abundance [12,41,50,52,].

Monitoring methods for both anadromous and landlocked alewife can be costly and labor intensive, and raise questions about whether other sampling methods may be more efficient. Several studies have targeted alewife with eDNA in recent years, often in the context of rivers and tributaries, and occasionally lakes [53, 54, 55, 56, 57]. To our knowledge, no study has attempted to use environmental effects as indicators of alewife presence, despite their well-recognized, strong effects on zooplankton communities and lake ecosystem [40,41,44]

In this study, we test the effectiveness and efficiency of direct sampling, environmental DNA, and measuring environmental effects for monitoring alewife of all life stages in small lakes in both non-native and restoration management contexts. We analyze each method using four metrics: 1) the ability for each method to accurately detect the presence/absence of alewife in small lakes, 2) the ability for each method to accurately detect the relative abundance of alewife in small lakes, 3) the cost efficiency of each method, and 4) the labor efficiency of each method. Understanding how each method scores along each metric is important for managers to make informed decisions about which methods to use in their particular contexts. Results of this comparison also provide insights into the promise and limitations of traditional sampling, eDNA, and environmental effects sampling for keystone species.

### Materials and methods

We sampled 30 small (<800 ha, mean = 102.7 ha) lakes along an alewife density gradient in August and September, when alewife juveniles were abundant in lakes, between 2018 and 2020 (Table 1). In total, we sampled 14 lakes with landlocked alewife populations, two lakes with anadromous alewife populations, and 15 lakes with no alewife. The number of lakes studied varied with each method based on cost and feasibility of study: direct sampling via purse seine (2 anadromous, 14 landlocked, and 3 non-alewife lakes), indirect sampling via eDNA (1 anadromous, 5 landlocked, 2 non-alewife lakes), and indirect sampling with environmental effects (2 anadromous, 13 landlocked, 15 non-alewife lakes). Lakes were chosen based on the historical record of alewife presence [40, 41; Michigan DNR personal communication). Through our study, two historic landlocked alewife lakes in Connecticut (Beach and Uncas) were determined to no longer contain alewife. From this data, we compare each method’s usefulness based on cost, time, and accuracy in determining presence/absence and abundance.

**Table 1:** Study lakes with location, basic characteristics, and alewife form. Data from direct sampling (alewife density), environmental DNA, and environmental sampling (zooplankton mean length) is listed for each lake. “Anad” refers to an anadromous life history, and “Land” refers to a landlocked life history. Letters “NS” represent “not sampled.”.

						Alewife	Lake	Max	Secchi	Surface	Alewife	eDNA	Zooplankton
Lake	State	Lat	Long	Month	Year	Life History	Area (ha)	Depth (m)	(m)	Temp (C)	Density (m2)	Total Hits	Mean Length (mm)
Bride	CT	41.326	-72.238	Aug	2018	Anad	18.2	8.6	2.5	29.1	3	5/9	0.32
Bride	CT	41.326	-72.238	Oct	2018	Anad	18.2	8.6	1.8	12.3	0.71	8/10	0.29
Bride	CT	41.326	-72.238	Nov	2018	Anad	18.2	8.6	2	5.3	<0.01	0/10	0.37
Dodge	CT	41.328	-72.198	Sep	2020	Anad	12.1	14.9	5	19.4	3.17	NS	0.41
Pigeon	MI	42.902	-86.204	Aug	2018	Land	91.1	7.6	3.9	24	0.61	1/10	0.28
Betsie	MI	44.63	-86.233	Aug	2018	Land	117.2	12.0	2.5	24.4	0.21	6/10	0.35
Paw Paw	MI	42.206	-86.27	Aug	2018	Land	346.8	27.4	2	25.6	0.14	7/10	0.32
Dumont	MI	42.593	-85.861	Aug	2018	Land	87.0	15.0	1	25.6	0.05	0/10	0.31
Pere Marquette	MI	43.941	-86.446	Aug	2018	Land	224.0	14.0	2.5	24.9	0.02	0/10	0.35
Woodland	MI	43.709	-85.861	Aug	2018	Land	82.2	15.5	2.5	26.8	0.12	0/10	0.37
Long	CT	41.446	-71.947	Sep	2020	Land	33.2	20.1	5.5	20.8	<0.01	NS	0.36
Pattagansett	CT	41.374	-72.229	Sep	2019	Land	49.2	9.5	3.3	25.1	0.09	NS	0.34
Avery	CT	41.493	-71.979	Sep	2020	Land	20.5	3.2	1	19.2	0.09	NS	0.31
Amos	CT	41.516	-71.975	Sep	2020	Land	42.0	14.2	1.8	21.4	0.09	NS	0.30
Rogers	CT	41.355	-72.298	Sep	2020	Land	105.3	18.6	3.3	25.8	0.12	NS	0.29
Quonnipaug	CT	41.393	-72.697	Sep	2019	Land	39.9	14.0	2.8	21.1	0.14	NS	0.39
Lime	MI	44.897	-85.837	Aug	2018	Land	271.1	20.4	2.3	25	0.04	NS	0.36
Uncas	CT	41.374	-72.315	Sep	2020	None	68.9	8.8	5	26	0	NS	0.51
Linsley	CT	41.317	-72.784	Sep	2019	None	9.3	14.5	2.8	22.8	0	NS	0.63
Beach	CT	41.578	-71.795	Sep	2020	None	140.1	19.3	5.5	20.4	0	NS	0.76
Miner	MI	42.567	-85.79	Aug	2018	None	131.5	25.3	2.8	25.4	0	0/10	0.67
Hackert	MI	43.983	-86.322	Aug	2018	None	50.6	11.5	5	26.7	0	0/10	0.89
Black	CT	41.525	-72.743	Sep	2020	None	23.2	6.3	2.5	25.7	NS	NS	0.42
Besek	CT	41.513	-72.732	Sep	2020	None	46.9	7.2	1.5	26.1	NS	NS	0.51
Wyassup	CT	41.488	-71.873	Sep	2020	None	40.1	8.3	3	21	NS	NS	0.58
Bashan	CT	41.491	-72.411	Sep	2020	None	110.5	13.6	4.3	24.8	NS	NS	0.67
Hayward	CT	41.521	-72.329	Sep	2020	None	70.5	10.4	2.3	24.6	NS	NS	0.67
Gardner	CT	41.508	-72.226	Sep	2020	None	19.5	8.2	2.3	24.6	NS	NS	0.70
Bitely	MI	43.742	-85.861	Aug	2018	None	14.6	6.5	5.5	26.3	NS	NS	0.93
Bear	MI	44.433	-86.152	Aug	2018	None	745.8	7.3	3.5	25.7	NS	NS	0.44
Fennessy	MI	42.952	-85.789	Aug	2018	None	62.5	2.5	2	26.3	NS	NS	0.70
Black	MI	42.062	-86.286	Aug	2018	None	8.1	7.0	2.5	24.4	NS	NS	0.76

*Traditional direct sampling—*We purse seined 15 lakes with known alewife populations (2 anadromous, 13 landlocked) with a purse seine that is 4.87m deep, 35.36m long, a mesh size of 1.57mm, and encircling an area of 100m^2^. We also purse seined two historical alewife lakes that no longer contain alewife after zooplankton results suggested that their alewife populations had been extirpated. We performed three inshore sets in less than 4.5m of water, and three offshore sets in deeper than 5m of water, for each lake sampled [see: 41, 43, 47, 53]. Each set was performed at least 30 minutes after dark, when alewife migrate up into the epilimnion and spread out to forage on zooplankton [58, 59, 60, 61; 62]. Our last set was always finished before 2:00am. If no alewife were detected, we put LED lights into the water, which attract alewife, and sampled again (see [63]) in order to detect their presence (and not their density). We counted and recorded the number of alewife caught within each set. To alleviate suffering, alewife that were caught in the purse seine were kept in the water and immediately released after counting. No alewife were sacrificed, and no anesthesia or analgesia were used for this study. From non-LED light sets, we estimated alewife density for each lake. All fish sampling was conducted under Yale University IACUC Protocol #2018–10734, State of Connecticut Scientific Collector Permit SC-17025 and State of Michigan Scientific Collectors Permit CT-177820047. All field sites were open to the public and accessed via publicly available boat launches and did not require permitting with the exception of Bride Lake in East Lyme, Connecticut. Access to Bride Lake was granted by York Correctional Institution in East Lyme, Connecticut (https://portal.ct.gov/doc/facility/york-ci).

*Environmental DNA—*We sampled eDNA from 7 alewife lakes and 2 non-alewife lakes. All of the lakes were sampled in August of 2018 along the western coast of Michigan, with the exception of Bride Lake, which is located in coastal Connecticut. Before sampling a lake, the entire boat was sprayed with a 10% bleach solution and air dried to decrease risk of contamination [64]. When more than one lake was sampled on the same day, the lakes were sampled on a gradient from lowest to highest density of alewife. We sampled Bride Lake, a lake with anadromous alewife, in August, October, and November of 2018 as alewife left the lake to test for the responsiveness of eDNA to changes in alewife density. Each sampling with eDNA was paired with density estimates obtained by purse seining as young-of-year anadromous alewife migrated back to the Atlantic Ocean.

Within each lake and on each sampling date, we took ten (10) replicates of 355 ml surface water samples with PET plastic short disposable water bottles. We chose 10 replicates per lake based on previous success with a different species and for cost effectiveness [20]. The 355ml sample volume was chosen based on availability of the containers and transportability in the field. For each lake, we collected five samples over 1m of water (inshore), and five samples over the main basin of the lake (offshore). Both inshore and offshore locations were sampled to account for alewife habitat preferences [65]. Inshore locations were evenly spaced around the lake, irrespective of bottom composition, with the first location a distance of ~50m away from a boat launch. Offshore locations were evenly spaced over the lake’s basin, irrespective of bottom composition. After filling each bottle, we placed the bottles on ice in a cooler washed in bleach solution to prepare for filtering. A bottle full of tap water was stored in the cooler during field sampling and used as a field blank.

Within 8 hours of collecting the water samples, each sample was filtered through a 1.2um Whatman Glass Microfiber (GFF) filter [20; 54] in a 250mL Nalgene 145–2045 polypropylene analytical test filter funnel and placed in a 2ml centrifuge tube filled with cetyl trimethylammonium bromide (CTAB). Tap water was run through a filter as a filter blank to ensure the filters and funnels were free of contaminants. Samples were then frozen for long term storage. Samples were sent to the University of Maine for primer validation, DNA extraction, and qPCR testing.

The eDNA assay used for this study was developed as part of the *University of Maine eDNA Toolkit for Northeast Diadromous Fishes* developed in support of a NOAA Fisheries contract to develop a compatible, standardized, and reconfigurable eDNA assay toolkit for 10 diadromous fishes (S. Silverbrand, G. York, and M. Kinnison pers. comm.). The assay was used to detect alewife DNA in our water samples after optimization runs with previously published alewife assays showed very late amplification of high standards [54]. In order to validate the alewife specific primer set developed in New England for use in other regions, DNA was isolated from Lake Michigan alewife muscle tissue using DNeasy Blood & Tissue kit (Qiagen, Valencia, CA, USA). qPCR reactions were run on a Bio-rad CFX-96 using the primer specific protocol. DNA isolated from New England derived as well as Michigan derived fish were normalized and run to compare efficiency across regions. Our resulting primer pair was: forward 5’-TTGGCTCAACCAAAACTATCACCCTCA-3’ & reverse: 5’-ACGGTGGCAGTGAGAGGAAATCCC-3’. Our probe was: VIC-TGCCTTATTCTTCTCTCCTTAGGAGGC-MGBNFQ. Standard curves were created using gBlock Gene Fragments run independently in the same lab, and were used to establish qPCR efficiency and estimate DNA starting copy number for the environmental samples.

Filtered water samples were stored at -20^0^C until extraction, which was completed using an altered DNeasy Blood & Tissue kit (Appendix X). Possible PCR inhibition was removed from samples using Zymo OneStep PCR inhibitor Removal Kits (Zymo Research, Irvine, CA, USA). qPCR tests were performed on a Bio-rad CFX-96 using the following thermal protocol: (95^0^C for 10 minutes, (95^0^C for 15 seconds, 60^0^C for 15 seconds) x 50 cycles). The reaction chemistry was as follows: 10 µl Taqman Environmental Master Mix 2.0, 3 µl template DNA, assay concentrations of 1µM primers, 500nM probe, and nuclease free water to bring the reaction volume to 20ul. All samples, controls, and standards were run with four technical replicates.

After gathering qPCR results, we investigated the detection probability of alewife using a simple single-season hierarchical occupancy model [66]. False negatives, or a failure to detect eDNA in the known presence of an organism, are common in eDNA methods [67]. Detection probabilities can help researchers better refine their methods for more efficient use of eDNA technologies. We did not subject other methods to hierarchical occupancy models because they had 100% detection rates. We tested 8 covariates of alewife presence in our occupancy model: secchi disk depth, surface dissolved oxygen, surface temperature, lake area, maximum lake depth, total alewife density, inshore alewife density, and offshore alewife density. The model was performed through PRESENCE software (v. 2.13.42) from USGS.

*Environmental effects: Zooplankton Community Size Structure***—** We sampled 15 alewife and 15 non-alewife lakes for zooplankton community size structure, a method we propose as an indicator of alewife presence. Using bathymetric maps, we identified the deepest hole in each study lake and took one vertical zooplankton tow with a 30 cm diameter, 90 cm long, 80-um mesh plankton net. Samples were condensed in an 80-um mesh condenser cup and immediately preserved in 70% ethanol. In the laboratory, we split the zooplankton down to between 200 and 400 cladocerans and copepods (including nauplii) with a zooplankton splitter. We identified zooplankton to taxonomic family and measured the length of 200 specimens or the entire subsample, whichever came first.

We took the mean body length of cladocerans and copepods (excluding nauplii) as a measure of the alewife’s environmental effects. From these data and historical knowledge of alewife presence, we determined a specific mean zooplankton length to serve as a delineation point, by which we could determine alewife absence or presence. A mean zooplankton length above the delineation point would indicate the absence of alewife, and a mean zooplankton length below the delineation point would indicate the presence of alewife. Our criterion for selecting a delineation point was to minimize overlap of mean zooplankton lengths between alewife and non-alewife lakes, which serves to minimize false negatives and false positives.

*Financial and Time Cost Calculations*—We calculated the financial and time costs of traditional direct sampling, eDNA sampling, and environmental effects sampling. The calculations were based on the perspective of a field biologist that would need to contract a molecular lab to perform genetic analyses. Both financial and time costs could differ in different contexts depending on the type of equipment needed and the expertise of the researchers performing the sampling. We did not include costs that were shared by all three methods in our analyses. For example, boat and motor costs were not factored into financial costs, and travel time to and from lakes were not factored into time costs. We assume that access to laboratory space or field-garages were common to all methods. Labor costs were calculated with the assumption of $15/hour pay rate, which was listed as the mean fisheries technician wage in the state of Michigan in 2024 (ziprecruiter.com). We also assumed that all field work was completed by two individuals, following safe field sampling protocols. Costs were split up between non-consumable, one-time startup costs, and consumable costs that are dependent upon the number of samples collected. Specific financial costs of equipment were determined by online pricing in July of 2022. For eDNA extraction, inhibition removal, and qPCR, we used pricing listed by the Maine-eDNA center at the University of Maine (umaine.edu/edna/). Time costs were calculated based on personal experience performing each method.

## Results

*Alewife Presence/Absence***-** Traditional direct sampling for alewife presence with a purse seine initially appeared to have an 85% detection rate, but this increased to 100% when follow-up sampling corroborated that alewife are now likely absent from two lakes that historically contained them. We successfully detected alewife presence in each lake with historical records of alewife with the exception of two: Beach and Uncas lakes in Connecticut (Table 1). Additional purse seining around LED lights in Beach and Uncas resulted in zero fish caught. Because other sampling methods were consistent with this finding, we determined that Beach and Uncas no longer contained an alewife population.

With eDNA, we observed a 51.7% detection rate with 3 false negatives out of a sample size of 7 lakes (1 anadromous, 6 landlocked) (Table 2). By false negative, we mean that after analyzing 10, 355 mL water samples, we did not detect any alewife in a lake with a known alewife population. Of the two non-alewife lakes sampled with eDNA, we observed no false-positives, or instances where eDNA was detected without any alewife present in the lake. As expected, all controls came back negative.

**Table 2:** Environmental DNA results show average copy number, total eDNA hits, and identifying false positives and negatives. We received three false negatives, and no false positives.

Lake	Alewife Presence	Alewife Density	Average Copy Number	Total Hits	FALSE
Miner	no	0.00	0.00	0/10	
Hackert	no	0.00	0.00	0/10	
Pere Marquette	yes	2.33	0.00	0/10	negative
Dumont	yes	4.50	0.00	0/10	negative
Woodland	yes	12.00	0.00	0/10	negative
Paw Paw	yes	13.83	1.25	7/10	
Betsie	yes	21.33	1.51	6/10	
Pigeon	yes	61.00	0.05	1/10	
Bride	yes	300.43	0.26	5/9	

**Fig 1:**
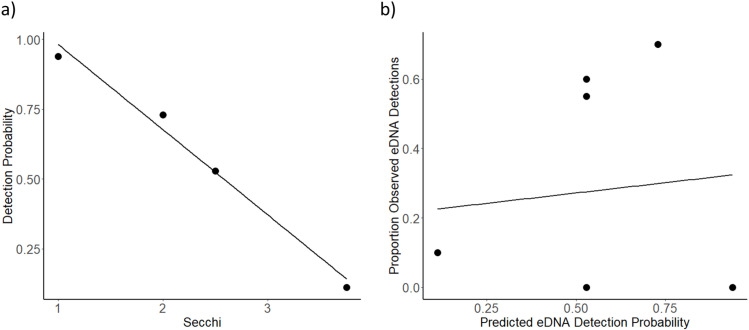
Heirarchical occupancy best fit model eDNA detection probabilities. Panel a) shows the model’s prediction that that as secchi disk increases, detection probability decreases. Panel b) shows the Predicted EDNA detection probability versus the observed EDNA detections.

A hierarchical occupancy model fit secchi disk depth as a covariate determining detection probability (AIC = 61.9) (PRESENCE version 2.13.42) (Table 3). The best fit model did not significantly correlate with observed detection probabilities (Regression, R^2^= < 0.01, F_1,5 _= 0.04, p = 0.84) (Fig 1). In Bride Lake, a lake with anadromous alewife that we sampled in August, October, and November as alewife migrated out of the lake, alewife eDNA was detected when anadromous alewife density was above 0.7/m^2^ in August and October, and was not detected after most alewife migrated out of the lake in November (<0.01/m2). Thus, eDNA effectively detected alewife DNA when in high abundances, but not at low abundances.

**Table 3:** Heirarchical occupancy model of EDNA detection probability and environmental covariates. Secchi disk depth (water turbidity) best determines detection probability.

Model	AIC	deltaAIC	AICwgt	Model Likelihood	P[1] Estimate
psi(.).p(Secchi)	61.9	0	0.3178	1	3
psi(.).p(Offshore Density)	62.35	0.45	0.2538	0.7985	3
psi(.).p(Area + Depth + Secchi+ Offshore Density)	63.05	1.15	0.1517	0.5627	6
psi(.).p(Secchi + Offshore Density)	63.75	1.85	0.126	0.3965	4
psi(.).p(Area + Depth+ Secchi + Offshore Density + DO)	65.05	3.15	0.0658	0.207	7
psi(.).p(Area+ Depth + Secchi)	65.18	3.28	0.0617	0.194	5
psi(.).p(Area+ Depth)	65.23	3.33	0.0601	0.1892	4
psi(.).p(Area + Depth + Secchi + DO)	65.68	3.78	0.0391	0.1511	6
psi(.).p(Depth)	66.8	4.9	0.0274	0.0863	3
psi(.).p(Area + Depth + Secchi + Temp + DO + Offshore Density)	67.05	5.15	0.0242	0.0762	8
psi(.).p(DO)	67.55	5.65	0.0189	0.0593	3
psi(.).p(Area)	68.55	6.65	0.0114	0.036	3
psi(.).p()	68.9	7	0.0096	0.0302	2
psi(.).p(Area+ Depth +Secchi + Temp + DO + Alewife Density)	69.68	7.78	0.0065	0.0204	8
psi(.).p(Surface Temperature)	70.18	8.28	0.0051	0.0159	3
psi(.).p(Total Alewife Density)	70.84	8.94	0.0036	0.0114	3
psi(.).p(Inshore Density)	70.85	8.95	0.0036	0.0114	3

**Fig 2:**
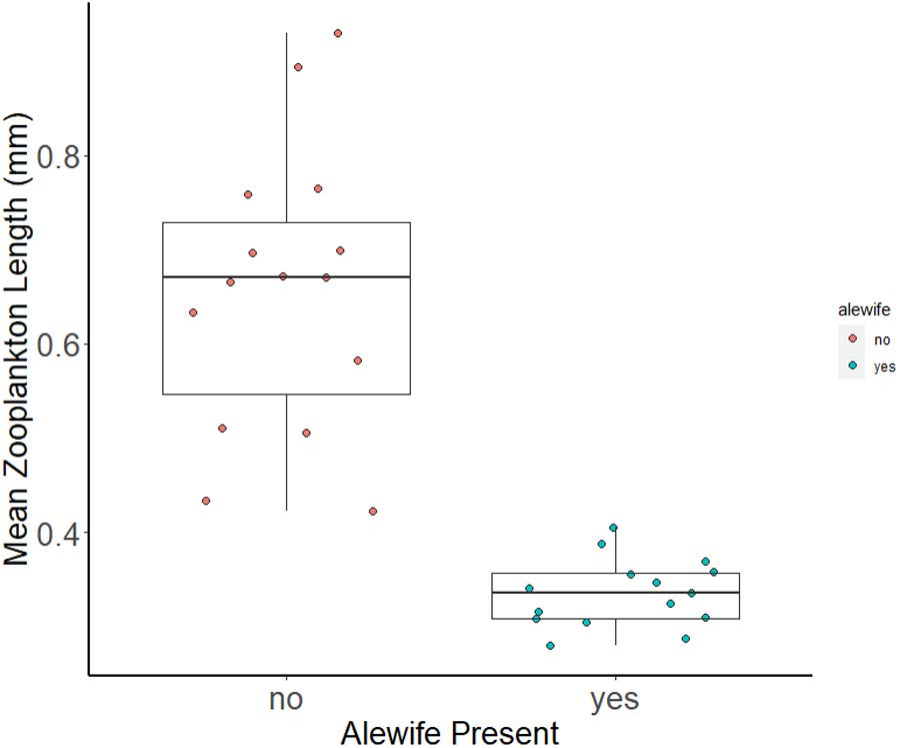
Mean zooplankton length (cladocerans and copepods, excluding nauplii) successfully detect alewife presence/absence with 0.42mm mean zooplankton length as the delineation point.

We observed a 100% detection rate identifying the presence and absence of alewife in lakes using environmental effects, or mean zooplankton length of crustacean zooplankton (cladocerans and copepods, excluding nauplii), in 30 lakes throughout Western Michigan and Eastern Connecticut (Fig 2). Zooplankton in lakes with alewife (Mean zooplankton body size = 0.34mm, SD = 0.036) and lakes without alewife (Mean zooplankton body size = 0.66mm, SD = 0.15) differed significantly in mean body length (t = 8.10, df = 15.60, p= < 0.001). We observed no overlap in mean zooplankton body-size between lakes with and without alewife. The maximum mean zooplankton body-size in alewife lakes was Dodge Lake, an anadromous lake in Connecticut with a mean zooplankton length of 0.405 mm. The minimum mean zooplankton body-size in non-alewife lakes was observed in Black Lake in Connecticut, with a mean length of 0.423 mm.

**Fig 3:**
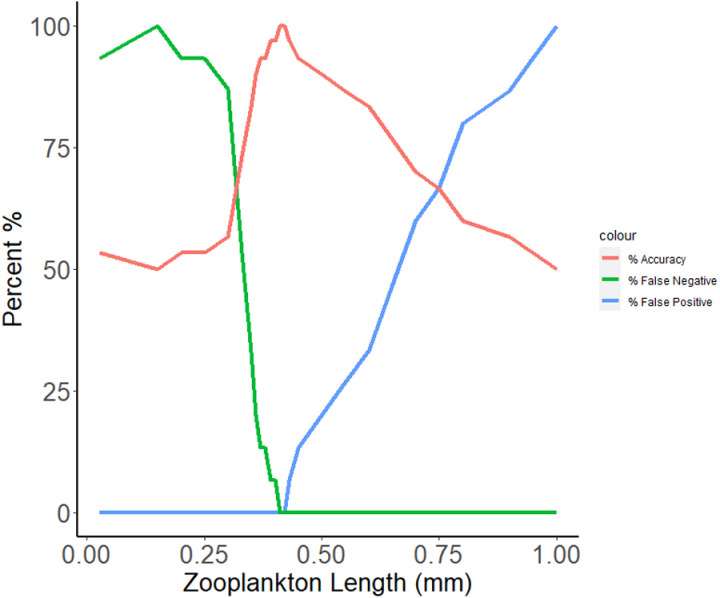
Zooplankton length and detection probabilities of the presence of alewife. A mean zooplankton body length of 0.415mm as delineation point produced the least number of false positives (Green—0) and false negatives (Blue—0).

Mean zooplankton body length provided a very useful and reliable determinant of alewife presence or absence based on detection probabilities determined from our sampling (Fig 2). A mean body length of 0.415mm provided the best delineation point for determining the presence and absence of alewife, resulting in zero (0) false positives and zero (0) false negatives. When the mean zooplankton body length delineation point was moved to 0.4 mm, we received one (1) false negative (the lake has a known alewife population, but the community size structure of the zooplankton sample suggests a lack of alewife) (6.67%). If moved to 0.38 mm, we received two (2) false negatives (13.34%). A delineation point of 0.43mm led to one (1) false positive (6.67%), and 0.45mm to (2) false positives (13.34%) (Fig 3).

*Alewife Density***-** Direct sampling by purse seine showed an alewife density gradient within our study lakes of <0.01/m^2^ to 3.17/m^2^, with an average of 0.51/m^2^. Inshore alewife densities ranged from 0.001/m^2^ to 6.67/m^2^, with a mean of 0.87/m^2^. Offshore alewife densities ranged from 0/m^2^ to 1.93/m^2^, with a mean of 0.31/m^2^. Lakes with anadromous alewife had significantly higher densities (M = 3.08/m^2^, SD = 11.72) than lakes with landlocked alewife (M = 0.14/m^2^, SD = 15.79) (t = 31.60, df = 1.63, p= < 0.01).

**Fig 4:**
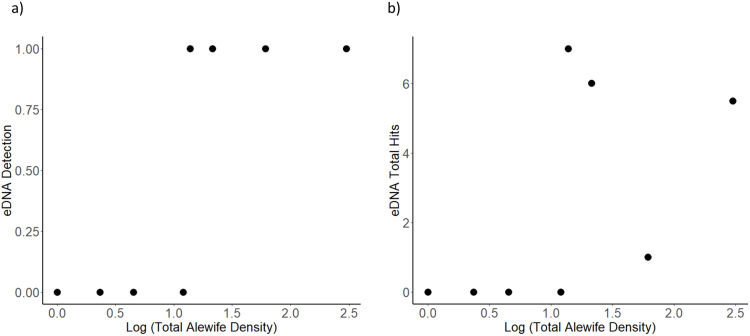
Environmental DNA detection (panel a: 10 samples either leads to alewife detection in a lake or it does not) and total hits (panel b: the number of detections out of 10) predicts alewife according to total alewife density (1 = present, 0 = absent).

Environmental DNA mean copy number and detection percentage did not significantly correlate with total alewife density (mean copy number, least squared regression, R^2^ = 0.08, F_1,7_ = 0.65, p = 0.45; detection percentage, least squared regression, R^2^ = 0.37, F_1,7_ = 4.13, p = 0.08). Inshore alewife density was significantly correlated with eDNA detection (least squared regression, R^2 ^= 0.50, F_1,7 _= 7.11, p = 0.03, slope = 2.46) but not mean copy number (least squared regression, R^2 ^= 0.13, F_1,7 _= 1.08, p = 0.33). Offshore density was not significantly correlated with eDNA detection (least squared regression, R^2 ^= 0.17, F_1,7_ = 1.47, p = 0.26) or eDNA mean copy number (least squared regression, R^2 ^= 0.07, F_1,7 _= 0.56, p = 0.0.48). No eDNA detections were observed in lakes with less than a 0.045m^2^ alewife density, and all lakes with an alewife density of 0.14/m^2^ had detections (Table 3; Fig 4). Alewife eDNA detections and average copy number was not significantly correlated with changes in density as anadromous alewife left Bride Lake (least squared regression, R^2 ^= 0.18, F_1,1 _= 1.17, p = 0.72).

**Fig 5:**
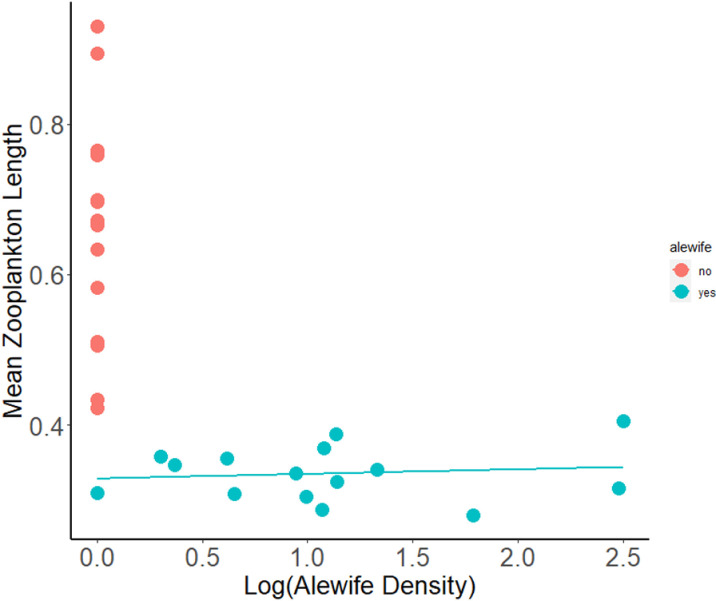
Environmental DNA detection (panel a: 10 samples either leads to alewife detection in a lake or it does not) and total hits (panel b: the number of detections out of 10) predicts alewife according to total alewife density (1 = present, 0 = absent).

Environmental effects of mean zooplankton body length were not significantly correlated with alewife density in lakes with landlocked alewife (least squared regression, R^2^ = 0.02, F_1,13_ = 0.20, p = 0.66, slope = 2.47). Alewife had a similarly large influence over zooplankton at low densities as at high densities (Fig 5).

**Fig 6:**
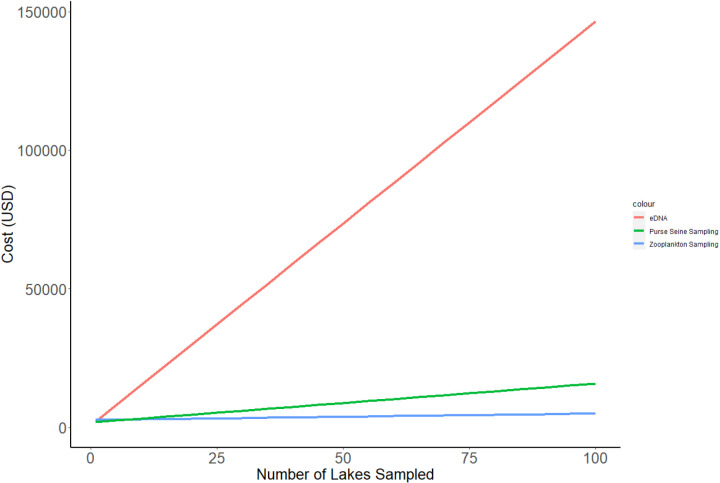
Financial costs required for monitoring 100 lakes with environmental DNA, environmental sampling by zooplankton sampling, and direct sampling by purse seine.

*Financial Cost***—** The cost of traditional, direct sampling by purse seine was in between environmental sampling and eDNA, with a one-time, non-consumable cost of ~ $1,800 (Table 4). A single 100m^2^ research purse seine was the bulk of the cost ($1,700). Other non-consumable costs include an 18-gallon bucket for anchoring the purse sein in the water (~$80), along with headlamps and a flashlight for effective purse seining at night when targeting alewife is most effective (~$60). Labor costs were $140 per lake for a total of 9.33 hours per lake (including 2 laborers) (Table 4). After sampling 100 lakes, purse seining costs a total of ~$15,820.92 (Fig 6).

**Table 4:** Financial costs of detection methods for alewife, including environmental DNA, environmental effects, and traditional direct sampling. Labor cost calculations were extracted from Table 5, and are similarly split between one-time and per-lake (consumable) costs.


Item	Consumable	Cost (USD)	Number	# Per Sample	Cost Per Sample (USD)
Traditional Sampling Costs					
100m research purse seine	no	1684.95	1		
18 gallon bucket	no	79.99	1		
Headlamp	no	19.99	1		
Flashlight	no	35.99	1		
Labor (0 hours)	no	0			
Labor (9.33 hours)	yes	140			23.33
**Environmental DNA Costs**					
Buchner funnel hand pump	no	59.99	1		
250mL disposable filter cups	no	485.07	50		
250mL bottles	no	62.49	12		
Bleach sprayer	no	9.97	1		
Plastic forceps	no	8.99	30		
GF/F 0.7um filters	yes	173	100	1	1.73
2mL centrifuge tubes	yes	36.93	500	1	0.07
CTAB buffer	yes	150	500mL	1mL	0.30
Pipette tips	yes	49.9	96	1	0.51
Latex gloves	yes	11.49	100	6	0.69
DNA extraction service	yes				8.50
Inihibition removal service	yes				6.25
qPCR	yes				25.00
Bleach	yes	14.02	3.78541	0.05	0.19
Labor (6.67 hours)	no	100			
Labor (6.83 hours)	yes	102.5			10.25
**Environmental Effects Costs**					
Zooplankton net	no	489.99	1		
500mL Lab wash bottle	no	20.99	1		
1/4“20m Rope	no	15.99	1		
Dissecting Microscope w/ scaled lens	no	1485.99	1		
Zooplankton condenser	no	5	1		
Zooplankton splitter	no	541.7	1		
Zooplankton counting platform	no	69.25	1		
4 oz Specimen container	yes	12.99	25	1	0.52
95% Ethanol	yes	32	3.8L	.08L	0.67
Labor (0 hours)	no	0			
Labor (1.48 hours)	yes	22.25			22.25

**Fig 7:**
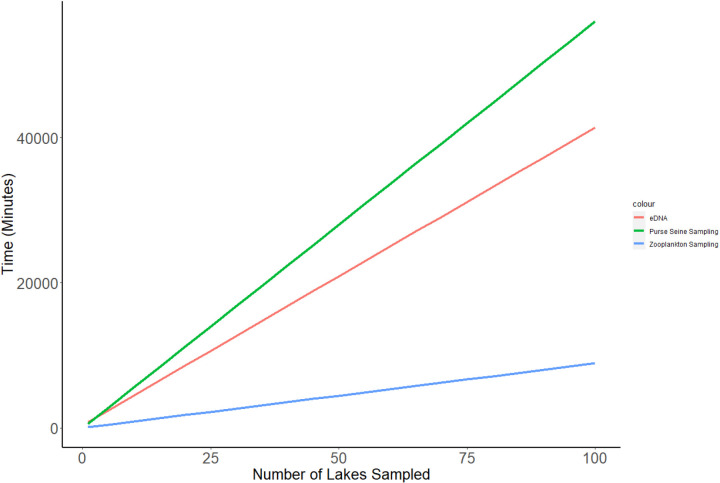
Time costs required for monitoring 100 lakes with each method: environmental DNA, environmental sampling by zooplankton sampling, and direct sampling by purse seine.

Environmental DNA was by far the most expensive sampling option for lakes with 10 samples per lake (Fig 6). One-time, non-consumable prices were the least expensive, costing $726.51 (Table 4). Non-consumable costs include 250mL disposable filter cups (which can be successfully re-used) (~$500), 250mL water collection bottles (~$70), and a Buchner funnel hand pump (~$60). However, consumable costs were ~ $45 per sample, with eDNA service costs through the University of Main costing ~ $40 per sample (Table 4). Labor costs for preparation, field-work, and analysis required a per lake cost of $102.50. After 100 lakes at 10 samples per lake, eDNA costs were estimated at $146,466.50.

Environmental effects sampling was the least expensive of the three methods after sampling 100 lakes (Fig 6). One-time, non-consumable cost of zooplankton sampling and analysis cost around $2,600. The highest laboratory cost was a dissecting microscope (~$1,500) and a zooplankton splitter (~$550) (Table 4). The most expensive field equipment cost was a zooplankton net, which costs around $500. Consumables for zooplankton sampling had a cost per sample of $1.19, which includes 25mL of 95% ethanol and one 100 mL specimen container (Table 4). Labor cost per lake was estimated at 1.48 hours total for two individuals, leading to a total labor cost of $25.25 per lake. After sampling 100 lakes, zooplankton sampling costs a total of $4,972.91 (Fig 6), more than 9 times less than the cost of using environmental DNA as an indicator of alewife presence.

*Labor Cost (time)—* The least time efficient of the three methods was direct sampling purse seining, which takes about 560 total minutes per lake for two individuals, or 56,000 minutes after 100 lakes (Fig 7). Before sampling, the 100m^2^ purse seine net needs to be dried and lightly bleached to prevent the transfer of invasive species (240 minutes) and the purse seine needs to be set properly in the boat for effective use (40 minutes per set). Sampling for density sets requires six purse seine hauls, which takes about 240 minutes. After sampling, the purse seine needs to be packed up for transport (40 minutes) (Table 5).

**Table 5:** Time costs of detection methods for alewife, including eDNA, environmental effects, and direct sampling. Tasks listed with a “Yes” under “One Time” are only counted for the first lake. Tasks listed with a “No” under “One Time” are per-lake costs.

Task	One Time	People	Time (min)	Total Time (min)
**Traditional Sampling Labor Time**				
Dry and bleach purse seine	No	2	120	240
Set purse seine in boat	No	2	20	40
Six sets	No	2	120	240
Pack up purse seine	No	2	20	40
**Environmental DNA Labor Time**				
Task	One Time	People	Time (min)	Total Time (min)
Prepare 2mL tubes/buffer	No	1	20	20
Collect 10 samples	No	2	60	120
Filter samples	No	1	40	40
Bleach sampling equipment	No	1	30	30
Specimen collection for assay design	Yes	1	200	200
Primer design	Yes	1	200	200
DNA extraction	No	1	100	100
qPCR	No	1	100	100
**Environmental Effects Labor Time**				
Task	One time	People	Time (min)	Total Time (min)
Single zooplankton tow	No	2	8	16
Bottle zooplankton	No	2	4	8
Rinse, split, and stage zooplankton	No	1	10	10
Count and measure 200 zooplankton	No	1	50	50
Data entry	No	1	2	5

The time commitment for preparing, collecting, and analyzing 10 samples eDNA was in-between the time commitment for environmental effects sampling and traditional sampling (Fig 7). One-time time costs included primer development, which in total takes around 400 minutes. The per-lake time commitment included a total of 410 minutes, which includes two individuals collecting 10 samples from different areas of the lake (120 minutes), and a single individual filtering the samples and putting them in a buffer for DNA preservation and transfer (40 minutes) (Table 5). Samples then need to be shipped off for DNA extraction and qPCR. After one lake, eDNA samples require about 810 minutes of time, or 41,400 minutes after 100 lakes.

Environmental sampling for zooplankton body-length required less time than both direct sampling and eDNA (Fig 7). Time costs include two individuals traveling to the deepest area of the lake and taking a single zooplankton tow (16 minutes), bottling the zooplankton for transfer to the lab (8 minutes), and a single individual splitting, counting, and measuring 200 individual cladocerans and copepods excluding nauplii (60 minutes) (Table 5). From entering the boat to calculating the average zooplankton community length (not counting travel time) takes about 89 minutes per lake, or after 100 lakes, about 8,900 minutes.

## Discussion

This study compared three methods for detecting the presence of alewife, a keystone species that is both a species of conservation concern and an invader, depending on the region. Our results demonstrate that measures of environmental effects can be a more effective and efficient means of biological monitoring than direct sampling with traditional methods or with eDNA. In the case of alewife in lakes, “environmental effects” means taking measurements of mean zooplankton length within the deepest part of the lake. Using a mean crustacean zooplankton length (excluding nauplii) of below 0.415 mm as an indicator of alewife presence (Fig 3), we predicted with a 100% success rate the presence or absence of alewife in 30 small study lakes (Fig 3). Through this study and its zooplankton length data, we discovered that two lakes which previously held alewife populations likely no longer contained alewife. Our initial findings were supported by extensive purse seining with LED pool lights, an effective method for determining alewife occupancy in small lakes (see [63]).

Challenges can emerge when quantifying a species’ environmental effects and determining whether the effects are indicative of the species’ presence. The alewife system has been well-studied for decades, and the alewife’s ecosystem effects have been quantified across their freshwater range [40,41,43–46]. The alewife’s strong interaction with their environment, and their ability to have large effects at low abundances, makes their environmental effects easily quantifiable. Keystone species that similarly interact strongly with their environments at low abundances are prime candidates for sampling with environmental effects [5; 68].

Alewife are not the only factor that can reduce zooplankton body size, however. Lake size, water temperature, toxicants, competitors, and other predators are all examples of drivers of zooplankton community size structure [69,70, 71]. While these factors did not influence the accuracy of environmental effects sample in our study lakes, the margin of error for determining alewife presence or absence was small. In our study, a 0.015mm increase or decrease of mean zooplankton length led to false positives or negatives (Fig 3). Determining a delineation point of environmental effect is therefore necessary before using environmental effect for management purposes (see Fig 3). Supplementing environmental effect sampling with direct sampling techniques can remove ambiguity when interpreting environmental effects. Due to the small margin of error and the plausibility of other factors leading to false positives or negatives, we suggest implementing supplemental direct sampling with environmental effects sampling. This supplemental sampling can only be determined by familiarity with the target species and their environmental effects. For alewife, we suggest supplementing environmental effect sampling with direct sampling when mean zooplankton length falls beyond 1.5 standard deviation below the mean zooplankton length in non-alewife lakes, and 1.5 standard deviation above the mean zooplankton length in alewife lakes. In our study, this represents values between 0.389mm and 0.432mm, and thus would require supplemental purse seining on 3 out of the 30 lakes, or 10% of lakes. When sampling 100 lakes, supplemental sampling would likely add 5,600 minutes to the work, and would cost an additional $3,220.92. In total, this would increase the time required to sample 100 lakes to 26,900 minutes (almost 27,000 fewer minutes than the next most time-consuming method) and a total monetary cost of $8,193.83 (which is $7,627.09 cheaper than the next most expensive method).

We also determined that environmental effect sampling was more cost effective and more time-efficient than other methods (Fig 6; 7), provided that presence/absence data are sufficient for the planned study. Non-consumable start-up costs are relatively low for zooplankton sampling ($2,628.91), with the majority of funds going toward a dissecting microscope, zooplankton splitter, and zooplankton net. Consumable costs are similarly low, costing only $1.19 dollars per lake (10 samples). These costs include only ethanol and disposable cups to store zooplankton samples (Table 4). Time costs for zooplankton are lowest among sampling methods, costing only 77 minutes per lake. Zooplankton collection requires a single zooplankton tow from the deepest part of a lake (total of 12 minutes for small lakes). The majority of time comes from laboratory analysis, which takes around 50 minutes. Zooplankton analysis does not require zooplankton identification, but only the measurement of 200 non-nauplii cladocerans and copepods (Table 5).

Zooplankton length was not an accurate predictor of alewife abundance (Fig 4). Alewife influenced zooplankton community structure similarly along an alewife abundance gradient of 0.005/m^2^ to over 3.1/m^2^. Power *et al.* [5] defined keystone species as species with a disproportionate impact upon its environment relative to its population abundance. Our finding that alewife environmental effects do not correlate with alewife abundance provides further evidence that alewife are a typical keystone species. It takes very few alewife to strongly structure the alewife community of lakes.

The most reliable method for documenting alewife abundance was direct sampling with a purse seine (Table 1). While purse seining is a relatively inexpensive and accurate tool for determining alewife density (Fig 6), each set takes about 30 minutes if a team is efficient, thus requiring considerable time on a lake to provide a robust population estimate (Table 5). However, to detect presence/absence of alewife, LED lights can be used in conjunction with purse seining to concentrate fish into the net, and this approach can reduce the number of sets required to confirm the incidence of alewife. While LED lights may allow for fewer sets, lights need to stay in the water for extended periods of time to function, and therefore may require the same amount of time and cost in the field [51].

Direct sampling alewife with purse seining offers a relatively efficient and effective means of detection. Initial, non-consumable financial costs for direct sampling were higher than environmental sampling and eDNA, but remained relatively reasonable at $1,820.92 (Table 4). Consumable costs of purse seining were cheapest among the three methods, making it an attractive option for sampling many lakes over long periods of time. However, time costs are the highest among the different sampling methods. Purse seining requires a team of skilled and able-bodied workers, requiring around 160 minutes per lake. Drying and lightly bleaching the purse seine is also required between lakes to avoid that transmittance of invasive species between lakes. Because of the purse seine’s size, this can take up to 120 minutes to clean and dry. Purse seining is only effective for less than 6m below the surface. Effective purse seining for alewife therefore occurs after dark, when alewife follow vertically migrating zooplankton to the epilimnion of shallow lakes (see [12,58–62]). Thus, purse seining requires work during non-business hours and contributes to its unattractiveness as an option for sampling many lakes over long periods of time.

eDNA was our least effective method at identifying the presence of alewife among all three methods. Out of 9 lakes, three came back as false negatives. No false positives were detected (Table 2). The reasons for the false negatives are not clear. Though based on a previously successful application of eDNA for a different species, our sample volumes may have been low, especially for the total lake size. In addition, though we targeted zooplankton where they were located, we did not use this same method for sampling eDNA, taking only surface water samples. In the summer, water columns stratify, and sampling may be more effective if targeted to where species are located or where the DNA containing material is settling out. Secchi disk depth, an estimate of water turbidity, provided the greatest explanatory power over our results (Table 3). Our higher occupancy model predicts that lower Secchi readings (or greater turbidity) may produce greater detection probabilities. The reasons for this could be that turbidity reduces light and UV penetration into the water column, which can destroy eDNA [20,27]. eDNA successfully determined that anadromous alewife migrated out of Bride Lake in November, after having been in large densities in October, suggesting that alewife eDNA did not persist in in surface water in Bride Lake for more than 35 days.

Our results after sampling 7 lakes with eDNA showed that neither average copy number nor hit percentage were reliable indicators of alewife abundance (Fig 4). eDNA successfully detected alewife in all lakes with an alewife density of 0.14/m^2^ or greater, and none below that density. Landlocked alewife are nearly exclusively pelagic fish that feed on large bodied zooplankton [65]. During the day, landlocked alewife may spend most of their time around the thermocline following zooplankton vertical diel migrations, which can be several meters deep in August and September, when this study was performed (see [58–62,65]). This means that any DNA left behind by alewife may be more concentrated in deeper water during the day. Sampling eDNA from below the water’s surface requires more effort or equipment or both to gain the same number of replicate samples and to minimize the risk of contamination between replicates. For example, Drummond *et al.* [72] used 3 Van Dorn samplers the required sterilization between lakes, and Monuki *et al.* [73] used multiple scuba divers for sampling kelp forests. Because we sampled for eDNA at the surface during the day, we may not have sampled deep enough for effective detection of offshore alewife (See [72,73]).

Anadromous alewife are often found in higher concentrations in shallower, inshore habitats [65] than landlocked alewife, which are predominantly pelagic. A reason for this difference in preferred habitat between landlocked and anadromous alewife may be their higher abundances in rearing lakes. When at high abundances, anadromous alewife decimate pelagic zooplankton populations which may push them inshore to feed in search of more plentiful littoral prey [74]. Anadromous alewife may also relate to inshore habitats more frequently than landlocked alewife due to subtle differences in development, which can lead to behavioral changes [65]. In Bride Lake, our only anadromous alewife lake sampled with eDNA, three out of five DNA hits were inshore location. Because anadromous fish are in shallower water, their DNA may persist on the surface for longer. More study is needed to understand where alewife eDNA is concentrated within a lake.

eDNA was the least cost efficient, and second least time efficient sampling method. eDNA had the cheapest up-front cost when samples are sent off for professional processing, but the cost of consumables for performing 10 replicates on each of 100 lakes would cost about 15 times that of sampling with a purse seine or zooplankton net (Fig 7). The time required to sample 100 lakes was not that dissimilar from traditional pure seining, but zooplankton sampling was by far the most time-efficient means of alewife detection. Because biological monitoring can require extensive sampling across large distances, the high cost with its relatively poor detection performance makes eDNA an less effective and efficient method for effective alewife monitoring compared to other methods in small freshwater lakes.

### Recommendation to ecosystem managers

We suggest that managers looking to monitor species with strong impacts on their environment, such as keystone species, consider whether measurements of ecosystem effects could be an effective and efficient means of determining the presence of the target species. Possible examples include monitoring Pisaster sea stars by the abundance of mussels, or sea otters by traits of kelp forests [35,36]. For monitoring alewife in small freshwater lakes, our example, we recommend that ecosystem managers use mean crustacean body-length (cladocerans and copepod excluding nauplii) as an indicator of alewife presence/absence. We recommend that mean zooplankton lengths between 0.389mm and 0.432mm be reinforced with targeted purse seining. This method both maximizes time and cost efficiency, while ensuring accuracy of detection. For alewife abundance, a combination of inshore and offshore purse seine is the most reliable method. The alewife’s effect on zooplankton community size structure has been extensively studied for decades, and across its freshwater range [40, 41, 43, 44, 45, 46]. We therefore recommend using zooplankton community size structure as an indicator of alewife presence throughout this same range. While environmental effects sampling was most effective alewife, a species with large ecosystem level impacts, it may not be effective for other species.
